# Mitochondrial redox systems as central hubs in plant metabolism and signaling

**DOI:** 10.1093/plphys/kiab101

**Published:** 2021-02-24

**Authors:** Olivier Van Aken

**Affiliations:** Department of Biology, Lund University, Lund, Sweden

## Abstract

Plant mitochondria are indispensable for plant metabolism and are tightly integrated into cellular homeostasis. This review provides an update on the latest research concerning the organization and operation of plant mitochondrial redox systems, and how they affect cellular metabolism and signaling, plant development, and stress responses. New insights into the organization and operation of mitochondrial energy systems such as the tricarboxylic acid cycle and mitochondrial electron transport chain (mtETC) are discussed. The mtETC produces reactive oxygen and nitrogen species, which can act as signals or lead to cellular damage, and are thus efficiently removed by mitochondrial antioxidant systems, including Mn-superoxide dismutase, ascorbate–glutathione cycle, and thioredoxin-dependent peroxidases. Plant mitochondria are tightly connected with photosynthesis, photorespiration, and cytosolic metabolism, thereby providing redox-balancing. Mitochondrial proteins are targets of extensive post-translational modifications, but their functional significance and how they are added or removed remains unclear. To operate in sync with the whole cell, mitochondria can communicate their functional status via mitochondrial retrograde signaling to change nuclear gene expression, and several recent breakthroughs here are discussed. At a whole organism level, plant mitochondria thus play crucial roles from the first minutes after seed imbibition, supporting meristem activity, growth, and fertility, until senescence of darkened and aged tissue. Finally, plant mitochondria are tightly integrated with cellular and organismal responses to environmental challenges such as drought, salinity, heat, and submergence, but also threats posed by pathogens. Both the major recent advances and outstanding questions are reviewed, which may help future research efforts on plant mitochondria.

## Teaser

Plant mitochondria are key components of redox homeostasis and play vital roles in regulating cellular metabolism, thereby affecting development and stress tolerance at the whole plant level.

**Advances**

- Improved quantitative MS-based approaches have accelerated the study of mitochondrial protein abundance, turnover and PTMs.
- Mitochondrial enzymes and cellular compartments operate interactively and efficiently exchange substrates.
- Roles for mitochondrial retrograde signaling in plant growth, during physiologically relevant stress conditions and in interaction with other organelles such as the chloroplasts, have been clarified.
- Further insights into mitochondrial antioxidant and peroxidase systems and how they affect other redox systems, enzymes, and whole plant growth have been generated.
- Our understanding of how mitochondria help plants power development and cope with adversity has improved.

## Introduction

**Figure 1:**
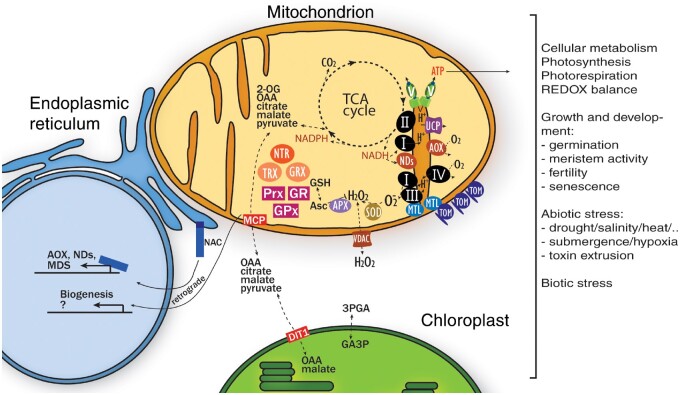
Plant mitochondria as hubs in redox metabolism, signaling, and plant growth. Plant mitochondria have a typical structure consisting of an outer membrane, inner membrane, and intermembrane space (IMS). The IMM forms large folds called cristae. The MTL complex is important for the formation of cristae at the cristae junction, and potentially interacts with TOM and mtETC components. The cristae lumen is thought to be important for the concentration of protons and protein complexes, improving metabolic efficiency. The TCA cycle uses substrates derived from glycolysis, photosynthesis, and amino acid metabolism to reduce NAD(P)+ to NAD(P)H. NADH is used to drive the mitochondrial electron transport chain (Complexes I–IV and AOXs and NDs), consuming O_2_. The cyt c pathway drives proton translocation from the mitochondrial matrix into the IMS, which can flow back via ATP synthase (green complex) to produce ATP or via UCPs without producing ATP. ATP can be exported into the cytosol and acts as a major energy source for a variety of cellular processes. Reductant from photosynthesis is transported to the mitochondria via the malate valve, which may be important to dissipate excess reductant from photosynthesis via the TCA cycle and mtETC. Excess citrate may leave the mitochondria via the citrate valve to be used in cytosolic metabolism. Other TCA cycle intermediates can be exchanged with the cytosol via mitochondrial carrier proteins to support metabolism. A TP-3PGA shuttle is thought to export NADH/ATP equivalents from the chloroplast into the cytosol. The mtETC inevitably produces superoxide as a by-product, especially under stress, which is rapidly scavenged by MnSOD, producing H_2_O_2_. H_2_O_2_ is further reduced in the Asc-GSH cycle, or by other peroxidase systems such as GPXs and Prxs. These peroxidase systems are regenerated by the Trx/ NTR system, ultimately using NADPH as a reductant. Mitochondrial GRXs mainly play a role in Fe–S cluster protein biosynthesis. Some of the H_2_O_2_ may either enter or leave mitochondria (via VDAC or aquaporins), which may have a signaling role. The functional status of mitochondria is communicated to the cellular nucleus via MRR. A key pathway that is activated during mitochondrial dysfunction is mediated by ANAC017-related transcription factors, which are anchored into the endoplasmic reticulum membrane. Upon stress, the NAC transcription factors are thought to be released by proteases and regulate gene expression of alternative mtETC components and other mitochondrial dysfunction stimulon genes. Other ANAC017-independent retrograde pathways are also likely to be active and may steer mitochondrial biogenesis. Overall, plant mitochondria act as redox, metabolism, and signaling hubs that affect all aspects of plant development and stress response.

Mitochondria most likely evolved by endosymbiosis of bacteria are related to alpha-proteobacteria with a host cell related to free-living Lokiarchaeota (Archaea) of the “Asgard” superphylum (Sagan, 1967; Spang et al., 2015; Martijn et al., 2018). This successful endosymbiotic interaction likely allowed the anaerobic host, previously fermenting organic substrates, to produce ATP far more efficiently using the endosymbiont’s aerobic respiration pathways (Roger et al., 2017; Seitz et al., 2019; Spang et al., 2019). Furthermore, adoption of these mitochondrial ancestors provided many additional biochemical pathways, giving the eukaryotic cell its metabolic flexibility. Over time, most of the endosymbiont’s genetic information was transferred to the nuclear genome. Surprisingly, most endosymbiont proteins no longer operate in mitochondria, but rather in the cytosol or elsewhere, or have been lost entirely. The complex mitochondrial proteomes are therefore mosaics of bacterial, host, and bacteriophage origin, along with new proteins that have evolved often in lineage-specific ways (Szklarczyk and Huynen, 2009, 2010; Huynen et al., 2013; Lama et al., 2019). Mitochondria have thus become an intrinsic and largely essential part of the eukaryotic cell, involved in energy production, biosynthesis, catabolism, and redox balancing. This requires mitochondria to be integrated into various sensing and signaling systems that affect the individual cell, but also the multicellular organism as whole. This review provides an update on our understanding of plant mitochondrial organization and the various ways in which plant mitochondria and their redox systems are involved in cellular metabolism, signaling, and plant life in general (Figure 1).

### Organization and structure of plant mitochondrial energy systems

The structure of plant mitochondria is relatively similar to that in other eukaryotes, and is crucial for how they function as efficient energy factories (Box 1; Friedman et al., 2015). Besides classic mitochondrial electron transport chain (mtETC) components like Complexes I–IV and ATP synthase (Complex V; Ikon and Ryan, 2017), plant mitochondria contain many components that are not present in many eukaryotes, such as alternative NADH dehydrogenases (NDs) and alternative oxidases (AOXs; Millar et al., 1994; Rasmusson et al., 2008; Vanlerberghe, 2013). Many other conserved mitochondrial complexes contain plant-specific subunits, such as respiratory complexes (Rao et al., 2017), and translocon of the inner/outer membrane (TIM/TOM) mitochondrial protein translocon complexes (Duncan et al., 2013) and mitochondrial ribosomes (Rugen et al., 2019; Waltz et al., 2019; Waltz and Giege, 2020). Other mitochondrial proteins are completely unique to plants or even individual plant families (Zhang et al., 2014; Lama et al., 2019), suggesting the plant mitochondrial proteome is still not fully fixed. In contrast, some plant species have surprisingly lost well-known mitochondrial components, like the hemiparasitic mistletoe (*Viscum sp.*), as found by sequencing the mitochondrial genome (Petersen et al., 2015). Biochemical characterization subsequently showed that mistletoe lacks a functional ND Complex I, while Complexes II, IV, and V also are divergent with much lower activities (Maclean et al., 2018; Senkler et al., 2018). In contrast, alternative mitochondrial pathway and cytosolic glycolysis activities were increased.

BOX 1 Mitochondrial organization and its effect on energy metabolism

Mitochondria are organized similarly in plants compared to most eukaryotes, with a double-membrane structure consisting of an OMM, intermembrane space, IMM, and a central matrix. The IMM usually has large folds forming the characteristic mitochondrial cristae. Cristae formation greatly enlarges the membrane area available to biochemical reactions such as the mtETC. Furthermore, it provides sub-compartments like the cristae lumen and cristae junctions with the IMM perimeter. These cristae compartments are crucial for maximal energy efficiency and concentration of protons, mtETC- and ATP synthase (super)complexes (Ikon and Ryan, 2017).

Abundant “non-bilayer” phospholipids like phosphatidyl-ethanolamine and mitochondrion-specific cardiolipin result in curved tubular membrane structures, which are likely important for cristae formation (Ikon and Ryan, 2017). “Mitochondrial contact site and cristae organizing system” (MICOS) protein complexes help demarcate these cristae junctions (Friedman et al., 2015).

In plants, loss of cardiolipin by mutating *cardiolipin synthase* results in various defects at the mitochondrial and whole plant level, including altered mitochondrial ultrastructure with unusual shapes and sizes, and fewer cristae (Pineau et al., 2013). Loss of cardiolipin resulted in a general loss of respiratory capacity, which could not be solely explained by reduced protein abundances, but rather pointed toward reduced overall mtETC efficiency (Petereit et al., 2017). In Arabidopsis, MICOS-related AtMIC60 interacts with OMM (e.g. TOM40) and IMM (e.g. Rieske iron–sulfur protein) proteins, and DGD suppressor 1 (DGS1), forming the “mitochondrial transmembrane lipoprotein” (MTL) complex (Michaud et al., 2016; Li et al., 2019). AtMIC60 appears to be involved in lipid trafficking, while the loss of DGS1 resulted in large mitochondria with fewer cristae and altered lipid composition, reduced protein import, and respiratory capacity (Michaud et al., 2016; Li et al., 2019). Various lipids and proteins thus help shape and support plant mitochondrial cristae, resulting in improved mitochondrial and plant performance.

This mitochondrial flexibility is to a large extent due to the plant alternative mtETC (NDs and AOXs; Millar et al., 2011). Although they do not translocate protons to drive mitochondrial ATP synthesis, they do allow a functional—if energetically more wasteful—electron transfer from, for example, NAD(P)H and succinate to oxygen (O_2_) when the cytochrome c (cyt c) pathway is compromised. While ND or AOX mutants tend to have mild phenotypic defects during optimal conditions (Giraud et al., 2008; Smith et al., 2011; Wallström et al., 2014a, 2014b; Wallström et al., 2014a, 2014b), they are affected when the cyt c pathway is inhibited (Dahan et al., 2014). Their effects are particularly noticeable in double mutants of cyt c and alternative pathway components, which perform poorer than the respective single mutants (Kuhn et al., 2015). If the alternative pathway cannot be induced sufficiently upon inhibition of the cyt c pathway, the plants rely more extensively on fermentation (Vanlerberghe et al., 1995; Wallström et al., 2014a, 2014b; Van Aken et al., 2016a, 2016b). Amino acids can also be directly used as substrates to maintain the mtETC during carbohydrate starvation (Cavalcanti et al., 2017), for example, via the electron-transfer flavoprotein:ubiquinone oxidoreductase (ETF/ETFQO) pathway located in the inner mitochondrial membrane (IMM) which is activated during senescence and drought (Pires et al., 2016).

Significant progress was made in our understanding of the plant Krebs/tricarboxylic acid (TCA) cycle, which appears to operate as a holistic process or “metabolon.” Using large-scale interactomics, 158 protein–protein interactions were identified involving TCA cycle components (Zhang et al., 2017a, 2017b, 2017c). These include interactions between subunits of sequential and nonsequential enzymes. Furthermore, substrate channeling of citrate and fumarate was shown, indicating a tight co-operativity between many TCA cycle enzymes, again likely to improve efficiency (Pineau et al., 2013; Petereit et al., 2017; Zhang et al., 2017a, 2017b, 2017c). TCA cycle intermediates can also fine-tune other mitochondrial proteins, like AOX isoforms that can be activated to various degrees by 2-oxoglutarate (2OG) and oxaloacetate (OAA; Selinski et al., 2018), as well as by pyruvate (Millar et al., 1993). Metabolites such as phosphoenolpyruvate and amino acids can stimulate (e.g. Pro and Ala) or repress (e.g. Lys) night-time mitochondrial respiration, involving Target Of Rapamycin kinase signaling (O'Leary et al., 2020).

Progress in proteomics now gives us much more detail into relative protein abundance (Rao et al., 2017; Fuchs et al., 2020), (super)complex composition (Senkler et al., 2017), and protein turnover rates (Li et al., 2017; Huang et al., 2020; Petereit et al., 2020). It is estimated that a single average mitochondrion contains >1.4 million individual proteins, covering up to 2,000 different types (Fuchs et al., 2020). Some proteins are extremely abundant, such as voltage-dependent anion channels (>40,000 units per mitochondrion), while others may be present less than once per mitochondrion (some RNA-binding pentatricopeptide repeat proteins). The TOM complex appears to be >20× more abundant than translocase of the IMM TIM22 and TIM23 complexes. In the IMM, ATP synthases are most abundant, followed by ADP/ATP carriers and Complex I. The mitochondrial matrix is heavily packed with TCA cycle enzymes (up to 16% of the matrix volume), which helps explain TCA cycle operation as a metabolon using substrate channeling (Zhang et al., 2017a, 2017b, 2017c). Individual proteins and complexes can range from <10 kDa up to supercomplexes of 1,500 kDa (I+III_2_ supercomplex). Some proteins are short-lived (e.g. less than a few days) while others may stay around for weeks (Nelson et al., 2013). These protein turnover processes are regulated by a wide set of mitochondrial proteases (Li et al., 2017; Heidorn-Czarna et al., 2018; Opalinska and Janska, 2018; Opalinska et al., 2018; Petereit et al., 2020). Together, this much-improved sense of scale is central to obtaining a realistic view of how mitochondria operate, are regulated, and maintained.

### Mitochondrial reactive O_2_ and nitrogen species production

Inevitably, electrons can leak from electron transport chains directly onto molecular O_2_, producing a wide variety of reactive oxygen species (ROS) including superoxide, hydrogen peroxide (H_2_O_2_), singlet O_2_, and hydroxyl radicals (Noctor and Foyer, 2016). In plant cells, various compartments contribute to ROS production, particularly the chloroplasts in illuminated conditions, the plasma membrane, apoplast, endoplasmic reticulum, peroxisomes, and mitochondria (Czarnocka and Karpinski, 2018; Smirnoff and Arnaud, 2019). These ROS molecules can damage cellular components such as DNA, lipids, and proteins, but also fulfill crucial signaling roles that help plants develop and deal with their everchanging environments. Therefore, the cell has evolved a wide range of antioxidant, sensor, and signaling systems to keep an appropriate redox balance.

In plant mitochondria mainly superoxide is initially produced, which is rapidly converted into H_2_O_2_by Mn superoxide dismutase (MnSOD; Blokhina and Fagerstedt, 2010). The half-life of H_2_O_2_ is much longer than that of superoxide, so superoxide is unlikely to traverse longer distances and act directly as an inter-organellar signaling molecule. Superoxide can directly damage Fe–S clusters, which are present in many mitochondrial enzymes, including Rieske iron–sulfur protein in Complex III, aconitase, and MnSOD itself (Morgan et al., 2008; Schwarzländer and Finkemeier, 2013).

The primary sites of superoxide formation in plant mitochondria are Complexes I and III (Juszczuk et al., 2012; Huang et al., 2016), each with different rates and topologies of production (Murphy, 2009). Both chemical and genetic inhibition results in overreduction of the mtETC, increasing the probability of electrons directly passing on to O_2_, producing superoxide (Meyer et al., 2009; Belt et al., 2017). Malate “circulation” from active chloroplasts to mitochondria (see the section on interactions of mitochondria with photosynthesis) can also trigger mitochondrial ROS production by, for example, Complexes I/III, and in excessive cases can even cause programmed cell death (PCD; Wu et al., 2015; Zhao et al., 2018), though the physiological importance under normal conditions is unclear.

Complex II (succinate dehydrogenase, SDH) also contributes to plant mitochondrial ROS production (Belt et al., 2017; Shin et al., 2020). Interestingly, low concentrations of salicylic acid (SA) increased Complex II ROS production, likely by stimulating SDH activity at or near the ubiquinone-binding site (Belt et al., 2017), while high SA concentrations may have an inhibitory effect on the mtETC (Norman et al., 2004; Poor, 2020).

Plant mitochondria also produce nitric oxide (NO) via reduction of nitrite by Complexes III/IV and AOX (Lazaro et al., 2013), particularly when O_2_ as electron acceptor is lacking (Gupta et al., 2011). NO can react with superoxide to form peroxynitrite, which is a highly reactive and potentially damaging molecule (Vandelle and Delledonne, 2011). Addition of nitrite and subsequent NO formation can, however, have a protective and ROS suppressive effect at low O_2_ levels (Gupta et al., 2011), by acting as an alternative electron acceptor to O_2_ and thereby maintaining membrane potential and ATP production (Gupta et al., 2017). Mainly the Q-cycle of mitochondrial Complex III is thought to generate NO, while AOX may reduce NO production by reducing electron flow through Complex III (Alber et al., 2017). A recent study further underlined that the way mtETC components contribute to NO production is very much dependent on the O_2_ availability (Jayawardhane et al., 2020). For instance, AOX was shown to prevent NO and superoxide production under normoxia, while under hypoxia it prevented superoxide generation and stimulated NO production. AOX was also found to be particularly important during reoxygenation following a hypoxia period by preventing nitro-oxidative stress. Overall, AOX appears to contribute positively to leaf energy status under various O_2_ levels.

### Mitochondrial antioxidant systems in plants

ROS can be both damaging and beneficial depending on their abundance, location, and timing. Therefore, mitochondria contain several enzymatic and nonenzymatic antioxidant systems, keeping ROS production within an optimal range by either scavenging ROS or preventing ROS production (Blokhina and Fagerstedt, 2010; Huang et al., 2016).

AOX gives the plant mtETC increased flexibility, allowing electron flow from reducing equivalents produced by the TCA cycle and photosynthesis to O_2_, even when the cyt c pathway is inhibited by stress, high membrane potential, chemicals, or mutations. Thus, the alternative respiratory pathway prevents excessive ROS production, rather than removing ROS that has already been produced (Cvetkovska and Vanlerberghe, 2013; Cvetkovska et al., 2014). When the basal alternative pathway capacity is superseded, AOX and ND components are rapidly induced at the transcriptional, protein, and activity level (Maxwell et al., 1999; Escobar et al., 2006; Van Aken et al., 2009; Vanlerberghe, 2013).

Mitochondrial uncoupling proteins (UCPs) provide an additional way to manage excess mtETC activity, by allowing protons to bypass ATP synthase across the IMM. UCPs play roles in thermogenesis, ROS homeostasis, and signaling, and regulation of energy metabolism (Barreto et al., 2016, 2017, 2020). UCPs are also important to facilitate efficient photosynthesis by maintaining mtETC redox poise (Sweetlove et al., 2006).

When ROS are produced in mitochondria, the first line of defense is the MnSOD, which converts superoxide into H_2_O_2_. MnSOD is among the most abundant proteins present in the mitochondrial matrix with around 10,000 units per average mitochondrion, in the same range as aconitase (Fuchs et al., 2020). Furthermore, MnSOD has a very high activity, indicating that MnSOD capacity in plant mitochondria vastly exceeds likely superoxide production rates by several thousand fold. This apparent excess may prevent direct damage of superoxide to Fe–S clusters present in many mitochondrial proteins (Halliwell and Gutteridge, 2015). Superoxide will therefore be converted almost instantly to H_2_O_2_, which thus is likely the most present ROS type in plant mitochondria.

It is, therefore, no surprise that many antioxidant systems in plant mitochondria prevent excessive H_2_O_2_ build-up. Ascorbate (Asc; vitamin c) and the tri-peptide glutathione (GSH) are crucial for mitochondrial H_2_O_2_ detoxification and redox balance (Foyer and Noctor, 2011). These seemingly distinct molecules operate together in the Asc–GSH cycle, where Asc reacts with H_2_O_2_ via Asc peroxidase (APX) to form monodehydroascorbate (MDHA). MDHA is also highly reactive and is neutralized using reduced GSH. The reducing power driving these reactions is provided by NAD(P)H, and at the end Asc and GSH are recycled, and H_2_O_2_ is neutralized to water. The GSH pool in plant mitochondria is kept in a highly reduced state under normal conditions (Schwarzländer et al., 2008) and is maintained relatively separate from the rest of the cell. For instance, it was found that despite 80% reduction of cellular GSH content in *phytoalexin-deficient pad2-1* mutants, the mitochondrial GSH levels were stable (Zechmann et al., 2008). During mitochondrial inhibition the GSH pool may be more oxidized, which could alter protein thiol redox state (Zsigmond et al., 2011).

A second mitochondrial peroxidase system uses GSH peroxidase (GPX)-like enzymes, which can scavenge H_2_O_2_ and other peroxides (Navrot et al., 2006). Despite their name, plant GPXs are not regenerated by GSH but by thioredoxins (Trxs; Marti et al., 2009; Yoshida et al., 2013). In Arabidopsis (*Arabidopsis thaliana*), one isoform GPX6/GPXL6 is targeted to mitochondria, but may be dual-localized in the cytosol (Attacha et al., 2017; Senkler et al., 2017). Rice (*Oryza sativa*) OsGPX3 is also mitochondrially targeted and is mainly expressed in roots, where it can be induced by cold and H_2_O_2_ (Passaia et al., 2013). Silencing *OsGPX3* resulted in a 20–30× higher release of H_2_O_2_ in rice roots (Passaia et al., 2013), indicating that GPX-like enzymes indeed play a significant role in peroxide removal from plant mitochondria.

Peroxiredoxins (Prxs) form a third major peroxidase system in plant mitochondria. Just like GPXs, they are thiol peroxidases that function in peroxide detoxification (Liebthal et al., 2018). The peroxidatic Cys_p_ is modified by the peroxide substrate to sulfenic acid, which can be subjected to further redox modifications such as sulfinic acid derivatives. These must be reduced by, for example, Trxs, sulfiredoxins, or NADPH-dependent Trx reductase (NTR) C to regenerate Prxs (Pulido et al., 2010; Iglesias-Baena et al., 2011). Interestingly, Prxs have also been implicated as redox sensors, and may act as primary ROS sensors (Liebthal et al., 2018). Plant mitochondria contain Prx IIF, which can form multimers and reduce a wide range of peroxides (Finkemeier et al., 2005). Under normal conditions, loss of Prx IIF function can be compensated by an increase in APX and GPX activity, but it appears to be important under stress conditions like CdCl_2_ excess and inhibition of AOX activity. In yeast (*Saccharomyces cerevisiae*), the mitochondrial Prx controls the oxidation state of GSH in the mitochondrial matrix in response to H_2_O_2_ that diffuses into the mitochondria from the cytosol via porins/voltage-dependent anion channels (Calabrese et al., 2019). Hyperoxidation of yeast Prx may function as an off-switch to limit mitochondrial GSH oxidation, thereby preventing cell death. A similar role for Prxs in plants has not been determined, so their role as a sensor or switch remains speculative.

To allow sufficient detoxification of peroxides, the above systems must be continuously regenerated. In plant mitochondria, this reducing power is provided by Trx/NTR and GSH/glutaredoxin (GRX) systems, which themselves are ultimately supplied with electrons from NAD(P)H (Box 2; Daloso et al., 2015; Ortiz-Espin et al., 2015; Riemer et al., 2015, Calderon et al., 2018). Our view of plant mitochondrial antioxidant systems has improved over the last years, showing it is a partially redundant network that can affect, for example, TCA cycle activity, the mtETC, and ROS production (Box 2).

BOX 2 Plant mitochondrial Trxs and GRXs

Trx/NTR and GSH/GRX systems regenerate the peroxidase systems, driven by NAD(P)H. Plant mitochondrial Trxs reduce a wide range of mitochondrial proteins, for example, Prxs, GPXs, AOX, and mtETC components, ATPase subunits (Marti et al., 2009; Yoshida et al., 2013). AOX activity is redox-regulated in vitro by Trx (Yoshida et al., 2013), but absence of Trxo1 does not reduce and actually increases AOX activation in vivo (Florez-Sarasa et al., 2019). Other thiol redox systems in the cell may compensate for Trx loss and keep AOX in its reduced state, rather than regulating its activity. Mitochondrial NTR A/B and Trxo1 are proposed as master regulators of TCA cycle enzymes, with some enzymes stimulated and others deactivated (Daloso et al., 2015), suggesting differential redox regulation.

These enzymes contain conserved cysteine residues, which makes them potential Trx targets. Trxs may post-translationally regulate the activity of mitochondrial enzymes like SDH, fumarase, and ATP- citrate lyase (Daloso et al., 2015). A relative increase in flux through the TCA cycle occurs in *trxo1* mutants, which may cause the increased AOX activity (Florez-Sarasa et al., 2019). Overexpression of Trxo1 in tobacco could alleviate H2O2-induced damage and maintained GSH redox state (Ortiz-Espin et al., 2015). Absence of mitochondrial Trxo1 did not lead to visible phenotypes under normal or salinity conditions (Calderon et al., 2018), while others reported alterations in Arabidopsis rosette size by 6 weeks of age (Daloso et al., 2015; Florez-Sarasa et al., 2019). Recent work shows that the CBS-domain containing protein CBSX3 interacts with Trxo2 and SDH1, and CBSX3 could stimulate Trxo2 activity (Shin et al., 2020). *CBSX3* overexpression led to increased ROS production, while *cbsx3* silencing and *Trxo2* knockdown surprisingly led to reduced mtETC ROS production (Shin et al., 2020).

Our understanding of mitochondrial GRXs also increased significantly. The mitochondrial monothiol GRXS15 is important for Fe–S protein maturation in Arabidopsis (Moseler et al., 2015; Ströher et al., 2016). GRXS15 can coordinate and transfer Fe–S clusters, powered by GSH, which is especially important for lipoic acid-dependent enzymes like GDC H protein. In contrast, GRXS15 has very low deglutathionylation and antioxidant reduction activity (Ströher et al., 2016).

GRXS15 loss-of-function plants displayed embryo-lethality or severe root growth reductions, showing that it has a crucial function most likely via its role in mitochondrial protein biogenesis, but unlikely for its potential role in antioxidant reduction (Moseler et al., 2015; Ströher et al., 2016).

### Interactions of mitochondria with photosynthesis, photorespiration, and central metabolism

Mitochondria do not function in isolation but form an integral part of a plant’s energy metabolism. Plant mitochondria are thought to act as a release valve for excessive reductants produced by the chloroplasts during photosynthesis, for instance under high light conditions, or during drought (Noguchi and Yoshida, 2008; Dahal et al., 2014). Especially AOX and NDs are thought to be important as they can dissipate excess energy without producing more ATP (Noguchi and Yoshida, 2008). The AOX pathway also plays a significant role in maintaining electron flow in the chloroplast ETC and reducing ROS production (e.g. via Asc) when the mitochondrial cyt c pathway is inhibited (Borisjuk et al., 2007; Benamar et al., 2008; Vishwakarma et al., 2015; Vishwakarma et al., 2018). AOX respiration could play an increasingly important role in carbon and energy balance under elevated CO_2_ conditions due to climate change, by preventing restriction of chloroplast ATP synthase activity (Dahal et al., 2017; Dahal and Vanlerberghe, 2018; Alber and Vanlerberghe, 2019).

An important part of this interaction between respiration and photosynthesis is the way reductant is shuttled from the chloroplast to the mitochondrion. A key component is the “malate valve,” in which malate is produced and transported out of the chloroplasts, where it can be consumed by malate dehydrogenases in the cytosol, mitochondria, and peroxisomes (Selinski and Scheibe, 2019). Such “malate circulation” to the mitochondria could regenerate NADH, which could drive Complex I but also contribute to mitochondrial ROS production, potentially even leading to PCD (Zhao et al., 2018; Zhao et al., 2020). A “citrate valve” is also potentially operating, where high NADH levels cause the Krebs cycle to operate as an incomplete “hemicycle.” Citrate is exported from the mitochondria into the cytosol where it is proposed to regulate NADPH/NADP^+^ balance, contributing to biosynthesis of amino acids and other compounds during photosynthesis (Igamberdiev, 2020). Together, the malate and citrate valves may balance the redox state of photosynthetic cells (Igamberdiev, 2020). The triose phosphate/3-phosphoglycerate (TP-3PGA) shuttle can also export ATP and NADH from the chloroplast to the cytosol (Shameer et al., 2019). Flux balance modeling suggested that mitochondrial ATP synthesis and export of chloroplast NADPH is also required to fulfill cytosolic ATP requirements even in daytime light conditions where chloroplast ATP synthesis is dominant (Shameer et al., 2019).

During photorespiration, where RuBisCO incorporates O_2_ instead of CO_2_ leading to photosynthetic losses, 3-PGA must be recovered by a complex interaction of enzymes in the chloroplast, peroxisomes, and also mitochondria. Mitochondrial glycine decarboxylase (GDC) converts glycine to serine during photorespiration, using NAD^+^ and releasing ammonia and CO_2_ (Douce et al., 2001; Araujo et al., 2014). The mitochondrial redox systems also impact on photorespiration function. Trx h2 (located in mitochondria and cytosol) regulates the redox status of the GDC L-subunit and can deactivate GDC-L in vitro (da Fonseca-Pereira et al., 2020). *Trx h2* mutants showed alterations in photorespiration and respiration metabolites, indicating an important role. Impairing mitochondrial *Trx o1* also resulted in restricted GDC activity under high-light conditions (Florez-Sarasa et al., 2019) and shifts from high to low CO_2_ (Reinholdt et al., 2019). These studies provide clear examples of how mitochondrial redox systems can directly affect mitochondrial and whole plant metabolism.

Plant mitochondria can also support cytosolic pathways. Methylglyoxal is a toxic by-product generated by glycolysis in the cytosol and is rapidly scavenged by the glyoxalase system, resulting in D-lactate production (Welchen et al., 2016). The mtETC accepts electrons from mitochondrial D-lactate dehydrogenase in the intermembrane space via cyt c, protecting against methylglyoxal and D-lactate toxicity (Welchen et al., 2016). The plant mtETC also plays a role in balancing cellular redox state in response to changes in nitrate/ammonium nutrition balance, for instance by affecting GSH redox metabolism (Podgorska et al., 2018; Rasmusson et al., 2020). Recent development of in vivo cytosolic NADH/NAD^+^ sensor lines using peredox-mCherry further showed that inhibition of the mtETC results in a gradual reduction of the cytosolic NAD pool, indicating that the cytosolic NAD redox state depends on mtETC activity for instance via external NDs or metabolite transport (Steinbeck et al., 2020).

All these interactions of mitochondria with cellular metabolism depend on the efficient transport of organic compounds, ions, and cofactors into and out of the mitochondria. A large family of mitochondrial carrier proteins is present on the IMM, which are primarily driven by the mitochondrial proton gradient and transmembrane potential (Lee and Millar, 2016). Substrates include acetyl-CoA, adenosyl nucleotides, TCA cycle intermediates, amino acids, and inorganic ions, but the directionality and specificity of each transporter protein have been a challenge to study. Novel in vivo sensor tools will be useful in obtaining more detailed insights (De Col et al., 2017; Wagner et al., 2019; Arce-Molina et al., 2020; Galaz et al., 2020). It is also unclear how they regulate or are regulated by redox signals and post-translational modification (PTM). The mitochondrial ATP/ADP carrier AAC1-3 was found to be acetylated, but the implications of this are unknown (Konig et al., 2014a, 2014b). A mechanosensitive channel-like protein, MSL1 was also shown to be targeted to plant mitochondria, where it acts as an ion channel to dissipate excessive transmembrane potential (Lee et al., 2016). *MSL1* can be induced during mitochondrial dysfunction (Van Aken et al., 2007), and under selected stress conditions loss of MSL1 resulted in a higher oxidation state of the mitochondrial GSH pool (Lee et al., 2016), suggesting it has a role in oxidative stress relief.

### Post-translational modification of mitochondrial proteins

Mitochondrial proteins are subjected to PTMs, which has been increasingly clear by improved proteomics methodologies as recently reviewed (Møller et al., 2020). PTMs on mitochondrial proteins include cysteine/tryptophan/methionine oxidation, nitrosylation, carbonylation, phosphorylation, lysine acetylation/succinylation, and more (Konig et al., 2014a, 2014b; Akter et al., 2015; Lu et al., 2018; Zhou et al., 2018; Møller et al., 2020; Nietzel et al., 2020). Such modifications are affected by kinases, (de)acetylases, ROS, and the mitochondrial redox systems described above, and can potentially change protein activity much faster than for instance transcriptional regulation. Classic examples like Cys disulfide bridges change protein structure and can affect metal ion binding. Phosphorylation introduces negative charges which can affect protein interactions, recognition, signaling, or affect activity, while acetylation neutralizes positive charges and adds hydrophobicity. PTMs on sensor proteins that act as on/off switches in redox and ROS signaling have been much sought after, but remain largely elusive (Liebthal et al., 2018). Despite the very long lists of PTMs on mitochondrial proteins, only few examples exist where these PTMs have a shown function, and a lot of the PTMs are considered “molecular noise” that arises chemically. It is not well understood if PTMs could act in a (semi-)quantitative way, where the number of PTMs per protein affects the function in a variable way. For instance, the oxidation state of AOX can be affected by Trx in vitro, but a lack of mitochondrial TrxO1 did not affect AOX redox state in vivo and even lead to an increase in activity (Box 2; Florez-Sarasa et al., 2019). In the case of GDC, redox-induced PTMs by Trx systems were shown to inhibit its activity resulting in in vivo effects (Reinholdt et al., 2019; da Fonseca-Pereira et al., 2020). In addition, many of the enzymes that add and remove these PTMs are still unknown (Konig et al., 2014a, 2014b). It will thus be a significant task to come to an accurate view of how PTMs dynamically modulate the various functions of plant mitochondria.

### Mitochondria-to-nuclear “retrograde” signaling

Most proteins operating in the mitochondria are encoded in the nuclear genome, so if there is a specific need to alter their production, the mitochondria must be able to relay this information to the nucleus. Furthermore, as mitochondrial function has far-reaching effects on the plant as a whole, retrograde signaling is used to regulate transcription of many nonmitochondrial proteins, thereby affecting plant growth and defense. An early observation of plant mitochondrial retrograde regulation (MRR) was the induction of AOX transcripts and protein in response to stress and inhibition of mitochondrial function (Maxwell et al., 1999). Many studies have now shown MRR occurs in response to chemical and genetic inhibition of mitochondrial function (Schwarzländer et al., 2012; Van Aken et al., 2016a, 2016b). Mechanistic insight was obtained into how plant MRR is controlled, by identification of mainly transcription factors that regulate or modulate expression of MRR target genes including ABI4, WRKY, MYB29, and NAC transcription factors (Giraud et al., 2009; Vanderauwera et al., 2012; De Clercq et al., 2013; Ng et al., 2013; Van Aken et al., 2013; Ivanova et al., 2014; Zhang et al., 2017a, 2017b, 2017c). Especially a class of membrane-bound NAC transcription factors plays a crucial role in plant MRR, with ANAC017 the most prominent in Arabidopsis (Van Aken et al., 2016a, 2016b). ANAC017 is anchored into the ER membrane, where it is cleaved upon mitochondrial dysfunction (probably by rhomboid proteases) and translocates to the nucleus to initiate expression of genes encoding mitochondrial (e.g. *AOX1a*) and nonmitochondrial (e.g. auxin glucosyltransferase *UGT74E2*) proteins (De Clercq et al., 2013; Ng et al., 2013). It operates in a positive feedback loop by activating similar genes like *ANAC013* (De Clercq et al., 2013), but is repressed by negative feedback from auxin signaling (Ivanova et al., 2014; Kerchev et al., 2014). Other factors like radical-induced cell death protein RCD1 bind the ANAC017-related transcription factors and repress their activity when not required (Shapiguzov et al., 2019). Recent work has shown that the ANAC017 pathway is most likely the functional equivalent of mitochondrial unfolded protein response-related (UPR^mt^) pathways that have been studied extensively in mammalian systems (Haynes et al., 2010; Pulido et al., 2010; Moullan et al., 2015; Quiros et al., 2017; Wang and Auwerx, 2017; Kacprzak et al., 2020). Although each eukaryotic kingdom appears to have evolved their own set of upstream regulators, the UPR^mt^ target genes have been well conserved, affecting mitochondrial functions such as chaperones, import components, and respiratory components, as well as systemic growth and defense regulators (Tran and Van Aken, 2020). MRR pathways also appear to overlap or interact with chloroplast retrograde pathways (Van Aken and Pogson, 2017; Pfannschmidt et al., 2020; Wang et al., 2020), and an ANAC017-independent MRR pathway may even control plastid gene expression in response to simultaneous inhibition of Complex IV and AOX (Zubo et al., 2014; Adamowicz-Skrzypkowska et al., 2020). Chemicals that inhibit mitochondrial function can trigger divergent MRR responses in light or dark conditions, indicating complex interactions also with chloroplast physiology (Alber and Vanlerberghe, 2019).

A key knowledge gap remains in how mitochondrial dysfunction is sensed. Many potential physiological parameters or second messengers that could be affected by mitochondrial function could be considered as players including ATP, NAD(P)H/NAD(P)^+^ ratio, Ca^2+^, pH, transmembrane potential, thiol switch proteins, PTMs, ROS, TCA cycle intermediates, oxidized peptides, etc. (Møller and Sweetlove, 2011; Vestergaard et al., 2012; Schwarzländer and Finkemeier, 2013). The clearest association is between MRR and H_2_O_2_, with many overlapping transcriptional responses between MRR and H_2_O_2_ treatment (Ng et al., 2013; Wagner et al., 2018). It is, however, unclear whether MRR and ROS production occur in parallel, or whether ROS formation is a key signaling component. Mitochondrial Ca^2+^ fluctuations are a less likely candidate, as mutants with defects in mitochondrial Ca^2+^ transport do not have typical MRR-related changes in, for example, AOX activity and resistance to Complex III inhibitor antimycin A (Wagner et al., 2015). Alternatively, one may argue that a lack of MRR-phenotypes in mitochondrial Ca^2+^ transport regulator mutants rather suggests that fluctuations in mitochondrial Ca^2+^ transport may be needed for MRR. With key regulators like ANAC017 residing in the ER, mitochondrial-ER contact sites may be of particular importance for MRR in plants (Michaud et al., 2016; Li et al., 2019; Liu and Li, 2019).

### Mitochondrial function from seed to senescence

The biochemical and signaling functions of mitochondria not only affect cellular homeostasis, but the impact on the whole plant. Many single or multiple mutants in nuclear genes encoding mitochondrial proteins have embryo-lethal phenotypes, underlining their essential role for plant viability (Carrie et al., 2010; Zhang et al., 2012; Moseler et al., 2015), even if some species have highly reduced mitochondrial functionality (Maclean et al., 2018). In the last 5 years, many more reports showed the importance of mitochondria both as energy factories and signaling components during plant development.

Shortly after seed imbibition, mitochondrial energy metabolism is crucial to move from a desiccated state into a functioning seedling with enough reserves to establish itself until the light is perceived and photosynthesis begins. Mitochondrial functions can already affect the sensitivity to germination by affecting abscisic acid (ABA) and gibberellic acid levels (Wang et al., 2014). Within minutes after imbibition, energy metabolism and respiration are started, mitochondrial transmembrane potential is established and ATP levels in the cytosol are increased (Paszkiewicz et al., 2017; Nietzel et al., 2020), well before transcriptional regulation could take effect (Law et al., 2012). The redox machinery also starts up very quickly, leading to rapidly reduced GSH pools in mitochondria and cytosol. This results in the reduction of cysteine residues in mitochondrial proteins belonging for example, to the mtETC and TCA cycle, which could act as thiol switches to regulate their activity (Nietzel et al., 2017; Nietzel et al., 2020). Especially the cysteines of NTRA/NTRB, GSH reductase (GR) 2, and Trx-o1 appear redox regulated, and mutants in these genes show delayed germination, suggesting redox regulation of the TCA cycle and mtETC plays an important role in efficient germination. When seeds have germinated under severe nutrient deficiency, they can remain in a stagnant phase for many weeks, from which they can recover upon nutrient supplementation (Rethore et al., 2019). Although cyt c mtETC capacity was the highest during the first stages of germination even under severe nutrient starvation, AOX capacity became dominant after several days during the transition to photo-autotrophy in low-nutrient germination conditions. Alternative mtETC activity, together with photorespiration, is thus likely to be important for energy dissipation from photosynthesis as well as maintaining mitochondrial carbon metabolism. Plant mitochondrial quality is also important for seed maturation and longevity, as mitochondrial mutants can show accelerated seed aging (Sew et al., 2016; Ratajczak et al., 2019). From a morphological perspective, mitochondria in dry and newly imbibed seeds are mostly spherical, but form a tubular network in the later stages of germination that partially envelopes the nucleus, which facilitates the mixing of mtDNA (Paszkiewicz et al., 2017). Mitochondrial motility thus increases once germination conditions are met, resulting in increased rates of mitochondrial fusion. When germination is completed, the mitochondrial network becomes more fragmented and heterogeneously distributed across the cell, in preparation for autotrophic growth of the growing seedling.

A recent study showed that mitochondrial function can affect apical hook formation during dark germination, which may protect meristems from damage during soil emergence (Merendino et al., 2020). ANAC017 and AOX1a play a role in this seemingly exaggerated ethylene response during germination, but it is unclear how it would work, as ANAC017 and ethylene signaling operate relatively independently (Wang and Auwerx, 2017; Kacprzak et al., 2020).

Plant meristem activity is also highly dependent on mitochondrial function, with many genes encoding mitochondrial proteins most highly expressed in meristematic tissues (Van Aken et al., 2007; Wang et al., 2019). These rapidly growing cells would have a high energy demand, so mutants in mitochondrial proteins concomitantly show reduced meristematic activity, often with underlying defects in mitochondrial morphology (Van Aken et al., 2007; Dolzblasz et al., 2018; Liu et al., 2019; Wang et al., 2019). These defects are often associated with increased ROS levels, deregulated auxin balance, and activation of MRR (Van Aken et al., 2007; Passaia et al., 2013, 2014; Yang et al., 2014; Kong et al., 2018; Liu et al., 2019). MRR may operate to repress auxin abundance and signaling for instance by induction of auxin-conjugating enzymes, affecting auxin transport (Tognetti et al., 2010; Ivanova et al., 2014; Liu et al., 2019) and through interactions of NTR/GSH pathways and auxin signaling (Bashandy et al., 2010; Passaia et al., 2014). Some results also suggest ROS production in mitochondria could affect developmental regulators such *Plethora 1/2* and ERF transcription factors to control meristem activity (Yang et al., 2014; Kong et al., 2018).

Mitochondria affect later stages of plant development including fertility via cytoplasmic male sterility, which has been used in plant breeding (Dewey et al., 1987; Bohra et al., 2016). A wide range of mutants in mitochondrial proteins also show reduced fertility, for instance by defects in pollen germination or anther dehiscence (Selles et al., 2018; Shin et al., 2020), which may involve mitochondrial Ca^2+^ regulation and mtETC ROS production. Retrograde signaling seems to be important to at least partially rescue fertility in plants with mitochondrial defects (Van Aken et al., 2016).

Recycling of stored resources is crucial to allow survival under dark periods or to provide energy reserves for seed production. Mitochondria appear to be part of this recycling until the last stages of leaf senescence (Keech et al., 2007; Chrobok et al., 2016). Under complete darkness, mitochondrial metabolism is needed to extend plant survival by using branched-chain amino acids and cell wall-degradation products to produce ATP (Law et al., 2018). Mitochondrial retrograde signaling can affect plant senescence, with observations that continuous mitochondrial stress can extend plant life span (Wang and Auwerx, 2017), although it is hard to differentiate between an overall growth retardation and true suppression of aging. ANAC017 has also been suggested to affect plant senescence, though contradicting reports showing senescence stimulating (Meng et al., 2019) or repressing roles (Kim et al., 2018) need to be clarified. Selective autophagy of mitochondria (mitophagy) is poorly understood, but it appears to be important for optimal resource recycling during senescence (Li et al., 2014a, 2014b; Broda et al., 2018). The balance between cellular survival and PCD also affects whole-plant growth and survival. Plant mitochondria are involved in PCD, though unlikely via cyt c release as observed in animal systems, with both protective and PCD-promoting properties likely involving mitochondrial ROS production (Bi et al., 2014; Zhang et al., 2014; Van Aken and Van Breusegem, 2015; Wu et al., 2015; Zhang et al., 2017a, 2017b, 2017c; Zhao et al., 2018; Zhang et al., 2020a, 2020b).

### Mitochondria and stress tolerance

Many genes encoding mitochondrial proteins are highly stress-inducible at the transcript and protein level (Van Aken et al., 2009), and many studies have shown that mitochondria can play a significant role in plant stress response and survival (Berkowitz et al., 2016). Drought and salinity tolerance are clearly impacted by mitochondrial function and signaling, with many studies highlighting the role of AOX (Giraud et al., 2008; Skirycz et al., 2010; Dahal et al., 2014). It is thought that AOX maintains respiration during drought, providing an electron sink needed for photosynthesis and preserving chloroplast ATP synthase protein levels and activity (Dahal et al., 2014). AOX also appears to reduce oxidative damage (for instance, protein carbonylation) in chloroplasts and mitochondria during extreme drought (Dahal and Vanlerberghe, 2017). An unknown signal from the photosynthetic ETC may coordinate AOX and light-harvesting complex (LHCB) amounts particularly during stresses (Dahal et al., 2017). Similarly, overexpression of *UCP1* in *Nicotiana tabacum* allowed to maintain higher respiration and lower H_2_O_2_ production during drought, while regaining water faster after rewatering (Barreto et al., 2017). Mitochondria affect stomatal function, for instance AOX may reduce NO levels that could act as a signal to close stomata (Cvetkovska et al., 2014). A functional alternative mitochondrial ETF/ETFQO pathway was found to improve drought tolerance by supporting electron transfer into the mtETC (Pires et al., 2016). The putative mitochondrial pyruvate carriers NRGA1 and MPC1 negatively regulate guard cell signaling in response to ABA, with *nrga1* and *mpc1* mutant plants displaying increased ABA sensitivity in guard cells and increased drought tolerance (Li et al., 2014a, 2014b; Shen et al., 2017). The authors suggest that increased pyruvate content in the mutants induces stomatal closure by activating slow-type anion channels, which require NADPH oxidases and ROS (Shen et al., 2017).

The Trx system may also contribute to drought resistance in plants, as *ntra ntrb* and *trxo1* mutants displayed improved recovery in photosynthetic capacity (Fv/Fm) after consecutive drought events (da Fonseca-Pereira et al., 2019). A number of ANAC017 MRR target genes can positively affect drought/salinity tolerance including auxin glycosyltransferase *UGT74E2* (Tognetti et al., 2010), outer mitochondrial membrane (OMM) ATPase *OM66* (Zhang et al., 2014), SA sulfotransferase *AtSOT12* (Baek et al., 2010), and *AOX1a* (Giraud et al., 2008). This indicates that MRR has a role also during water-stress responses (Ng et al., 2013; Bui et al., 2020; Meng et al., 2020), likely by affecting hormone balance.

During flooding and submergence mitochondria are affected by O_2_ deprivation, and mitochondrial signaling plays an important role in flooding tolerance (Box 3). Several O_2_ sensors have been suggested in plants including cysteine oxidases that oxidize N-termini of ERF-VII transcription factors via cysteine–sulfenylation, leading to their degradation via the N-degron pathway and thus repressing hypoxic responses (Abbas et al., 2015; White et al., 2018). A role for Complex IV in acute hypoxia sensing was proposed in mammalian systems, potentially via hypoxia-induced superoxide production leading to MRR signaling (Sommer et al., 2017). The potential role of plant mitochondrial components as O_2_ sensors thus needs further exploration.

BOX 3 The importance of retrograde signaling under physiological conditions

O_2_ deprivation directly inhibits mitochondrial function, which plants face during flooding or germination in compact and/or anoxic soils. Low O2 availability may directly inhibit Complex IV and AOX, although Complex IV has higher affinity for O2 (Millar et al., 1994). Gene expression changes to low O_2_ are highly similar to for example, Complex III inhibition (Wagner et al., 2018), affecting many ANAC017-dependent MRR target genes.

Physiological parameters like cytosolic ATP concentration, pH, and redox state respond very similarly to inhibition of the mtETC and low O_2_, indicating mitochondrial dysfunction is important for hypoxia response (Wagner et al., 2019).

Accordingly, ANAC017 is an important positive regulator of Arabidopsis tolerance to submergence (Bui et al., 2020; Meng et al., 2020). ANAC017-target genes were more methylated during adulthood in accordance with higher susceptibility of adult plants to submergence (Bui et al., 2020). ANAC017 operates in parallel with WRKY transcription factors that co-regulate gene expression of genes encoding mitochondrial proteins (Van Aken et al., 2013; Meng et al., 2020).

Simultaneous inhibition of cyt c and AOX pathways resulted in repression of chloroplast gene expression, which was also observed during hypoxia (Adamowicz-Skrzypkowska et al., 2020). Submergence and hypoxia increase fermentation and lactate formation (Barreto et al., 2016), which may be suppressed by ANAC017- signaling (Van Aken et al., 2016b). UCP1 overexpression in tobacco also induced hypoxia marker genes, suggesting a conserved response. Interestingly, priming with low antimycin A concentrations protect adult plants against flooding damage, most likely via MRR activation (Bui et al., 2020). How (and which) MRR target genes precisely protect against hypoxia damage requires further investigation. This may involve interplay between NO and ROS production via AOX and cyt c pathways, but both NO producing and repressing roles for AOX have been suggested under hypoxia and normoxia, respectively (Gupta et al., 2011; Alber et al., 2017; Gupta et al., 2017; Vishwakarma et al., 2018; Jayawardhane et al., 2020). Potentially, AOX drives ATP production and the hemoglobin-NO cycle via NO generation under anoxic conditions (Vishwakarma et al., 2018). NO produced under low O_2_ conditions is likely to inhibit Complex IV activity to avoid the tissue reaching severe anoxia, for instance in a germinating seed (Borisjuk et al., 2007; Benamar et al., 2008). O_2_ sensing and light perception are important for seedling establishment, so mitochondrial function and MRR may have an important role during soil emergence, which needs further study (Abbas et al., 2015; Merendino et al., 2020).

Mitochondria help plants to cope with heavy metal and toxin-induced stress. The mitochondrial GrxS15 partially protects plants from arsenic toxicity, most likely indirectly through its role in iron-cluster transfer (Ströher et al., 2016). Many mitochondrial membrane channels and transporters have also been linked to heavy metal resistance. Mitochondrial pyruvate carriers prevent cadmium accumulation in Arabidopsis by driving the TCA cycle and GSH synthesis, which in turn support ATP levels to drive Cd^2+^ efflux transporters on root epidermal cells (He et al., 2019). MSL1 also appears to be required to maintain mitochondrial redox balance and GSH levels after exposure to Cd^2+^ and high temperature (Lee et al., 2016).

Finally, several studies have shown the importance of mitochondrial factors during biotic stress. Overexpression of outer membrane AAA ATPase *AtOM66* caused increased sensitivity to PCD induction, resulting in resistance to biotrophic pathogen *Pseudomonas syringae*, but hypersensitivity to necrotrophic pathogen *Botrytis cinerea* (Zhang et al., 2014). Although the mechanism is unclear, the *AtOM66 OX* plants contained increased levels of SA, which could affect their pathogen response. Such increased levels of SA have also been observed in mutants of mitochondrial ribosome RPS10 (Adamowicz-Skrzypkowska et al., 2020). AOX can repress mitochondrial superoxide bursts after inoculation with *P. syringae* pv. *Phaseolicola* (Cvetkovska and Vanlerberghe, 2013). This mitochondrial ROS burst appears to be required but not sufficient to cause PCD via the hypersensitive response (HR). Surprisingly, the superoxide burst induced by *P. syringae* pv. *maculicola* and antimycin A was delayed in plants lacking AOX, suggesting that the mtETC is a target during HR. A link between *SDH1* expression and SA-based defense pathways has also been shown by several studies. SDH1, potentially via its ROS producing capacity, may contribute to SA production, which was suggested to improve resistance to a range of virulent bacterial and fungal pathogens (Gleason et al., 2011; Belt et al., 2017; Zhang et al., 2020a, 2020b). Several genes encoding mitochondrial proteins such as *OM66* and dicarboxylic acid carriers are also incorporated into JA-dependent touch- and wounding defense networks, but their role here is still unclear (Van Aken et al., 2016a).

## Concluding remarks

The last years have seen a tremendous increase in our understanding of the inner workings of plant mitochondria, how they function within whole-cell metabolism and how they help plants coordinate development and stress response. This is to a significant extent due to improved experimental methods, but also by building on increasing knowledge, and a very active research community. Fortunately, many key questions remain unanswered, which should provide interesting research challenges for years to come (see Outstanding Questions).

**Outstanding questions**

- What is the importance of the many functionally uncharacterized mitochondria-targeted proteins?
- How is mitochondrial dysfunction sensed and relayed to key transcription factors?
- What is the occurrence and significance of interaction sites with mitochondria and other organelles such as the ER?
- How is mitophagy regulated, when is it engaged, and what is its importance?
- How are specific mitochondria marked for mitophagy?
- Are there redox-regulated thiol ‘on/off’ switches that function as sensors, regulators and signaling components?
- What is the importance of the wide variety of PTMs, and what are the enzymes that add or remove them?
- How do plant mitochondria precisely affect development and stress tolerance?

## Notes

O.V.A. performed the literature survey and wrote the manuscript.

The author responsible for distribution of materials integral to the findings presented in this article in accordance with the policy described in the Instruction for Authors (https://academic.oup.com/plphys/pages/general-instructions) is: Olivier Van Aken (olivier.van_aken@biol.lu.se).
